# Male infant patient with a mesenteric cyst in the greater and lesser omenta: a case report

**DOI:** 10.1186/s12245-020-00282-0

**Published:** 2020-05-11

**Authors:** Rocio del Pilar Pereira-Ospina, Laura Catherine Montoya-Sanchez, Diana María Abella-Morales, Javier Yesid Pinzón-Salamanca, José Miguel Suescún-Vargas, Sergio Rueda-Martínez

**Affiliations:** 1 Instituto Roosevelt, Av. Cra. 4 Este No 17-50, Bogotá, 110311 Colombia; 2 Universidad del Rosario, Bogotá, Colombia; 3 Home Hospitalization Program Instituto Roosevelt, Bogotá, Colombia; 4 Andes University, Bogotá, Colombia; 5 Sabana University, Bogotá, Colombia; 6 Military University, Bogotá, Colombia

**Keywords:** Cyst, Mesentery, Pediatrics, Abdominal pain

## Abstract

**Background:**

Mesenteric cysts are intra-abdominal masses of congenital origin, which most frequently occur in children, with an incidence of approximately 1 case per 20,000 pediatric admissions. Its progression can be asymptomatic, and its diagnosis can be incidental. However, it usually occurs with symptoms such as nausea, vomiting, constipation, sensation of a mass, and/or diarrhea. The diagnostic imaging method of choice is abdominal ultrasound.

**Case presentation:**

Below, we present the case of a previously healthy 1-year-old male patient with nonspecific symptoms, who was referred to a tertiary hospital. The presence of a mesenteric cyst was detected at the end of the diagnostic approach.

**Conclusion:**

It is important to know these pathologies even though they are infrequent, because although they are benign masses by definition, they can lead to complications such as intestinal torsion, intestinal obstruction, and even peritonitis.

## Background

Mesenteric cysts are intra-abdominal masses which very rarely occur in children (the incidence is lower in children than in adults) [1, 2]. The first case was reported in the year 1507 by Benevienal and there have been very few cases reported in the literature since then [3]. The incidence is approximately 1 per 20,000 pediatric admissions [2].

The location of these cysts is more frequent in the mesentery of the small intestine, followed by the mesentery of the colon and the retroperitoneum [4]. Most of these cases are asymptomatic and are detected incidentally [5, 6]. However, symptoms such as abdominal pain, nausea, vomiting, palpable masses, constipation, or diarrhea may occur [7, 8].

The differential diagnoses of this entity include intestinal duplication cysts, diverticulitis, appendicitis, and intestinal obstruction and ovarian, choledochal, pancreatic, splenic or renal cysts. Torsion, rupture, or intestinal obstruction is among the complications that may occur [8–10].

Described below is the case of a previously healthy 1-year-old male patient who was referred with a diagnosis of acute diarrheal disease and in whom a mesenteric cyst in the greater and lesser omenta was identified.

## Case presentation

**Fig. 1:**
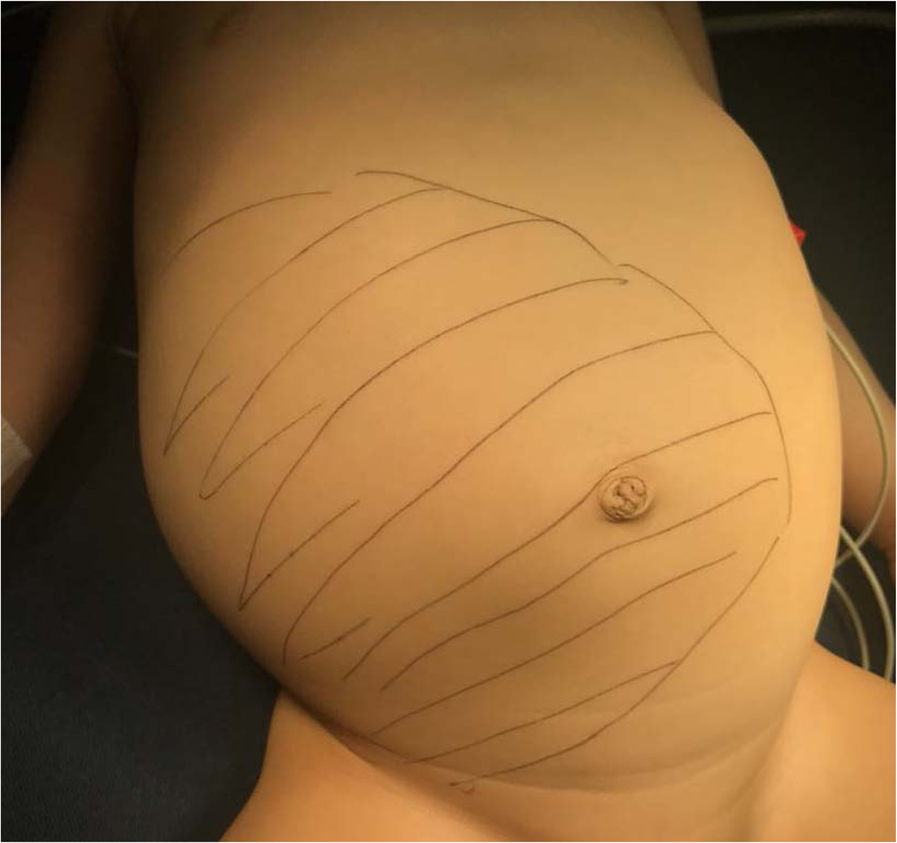
Delimitation of the abdominal lesion upon palpation

The patient is a previously healthy 1-year-old male patient who was referred to the institution because he had the following clinical symptoms for the past 15 days: multiple diarrheic stools with mucus and without blood (6–7 per day), associated with fever spikes, the highest being 39.8 °C; abdominal distension; and irritability. No additional symptomatology. Physical examination upon admission showed a patient in poor general condition, with marked abdominal distension and a palpable abdominal mass (Fig. 1), decreased bowel sounds, and generalized pain upon abdominal palpation. We decided to hospitalize the patient, with nil by mouth, with a nasogastric tube, and a request for an abdominal ultrasound and paraclinical tests.

**Fig. 2:**
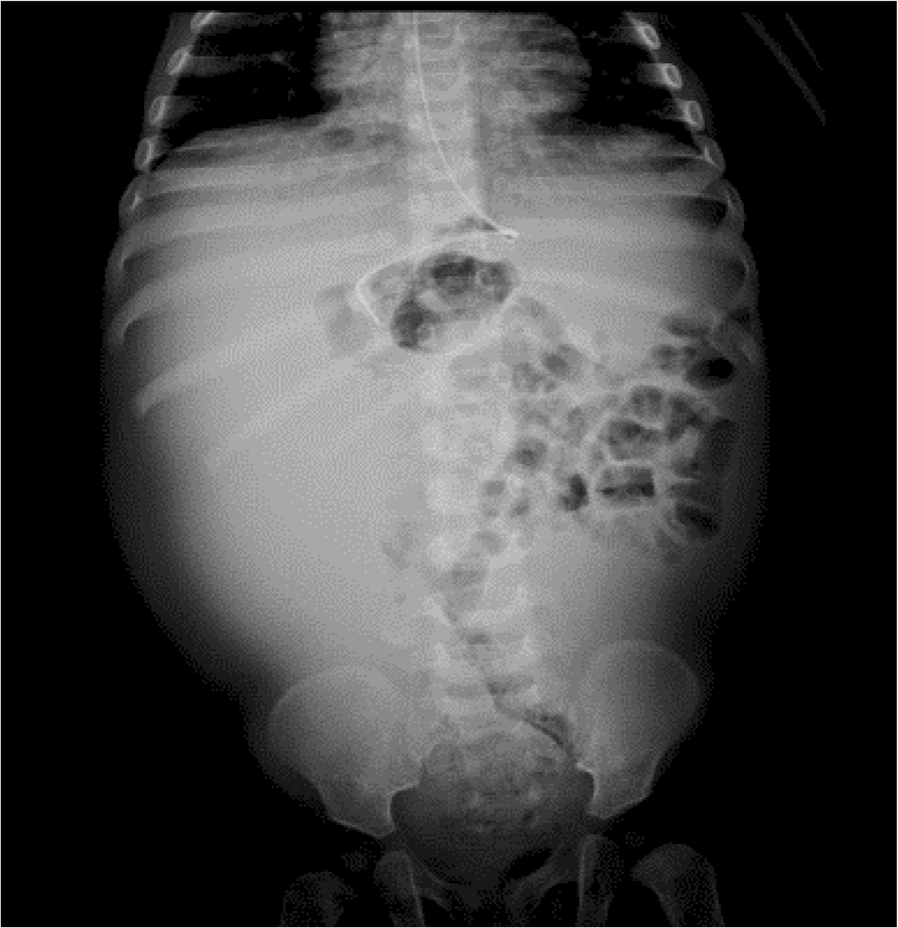
Bowel loops displaced to the left hemiabdomen, without evidence of hydroaerial levels, distal gas is seen. No extra-peritoneal gas or portal gas is observed. Opacification of the right hemiabdomen. There are no pathological calcifications

The paraclinical tests showed leukocytosis with neutrophilia and thrombocytosis, high CRP (97.1 mg/dL), mild hyponatremia (Na: 134 mmol/L), potassium, chloride, and calcium within a normal range; normal glycemia; kidney function within normal parameters; and coproscopic exam does not show parasitic structures. Abdominal ultrasound shows a multiseptate image which occupies the right half of the abdomen and displaces the bowel loops and could correspond to septate ascitic fluid; distension of intestinal loops. X-ray of the abdomen where displacement of the bowel loops to the left and opacification of the right half of the abdomen is seen (Fig. 2).

**Fig. 3:**
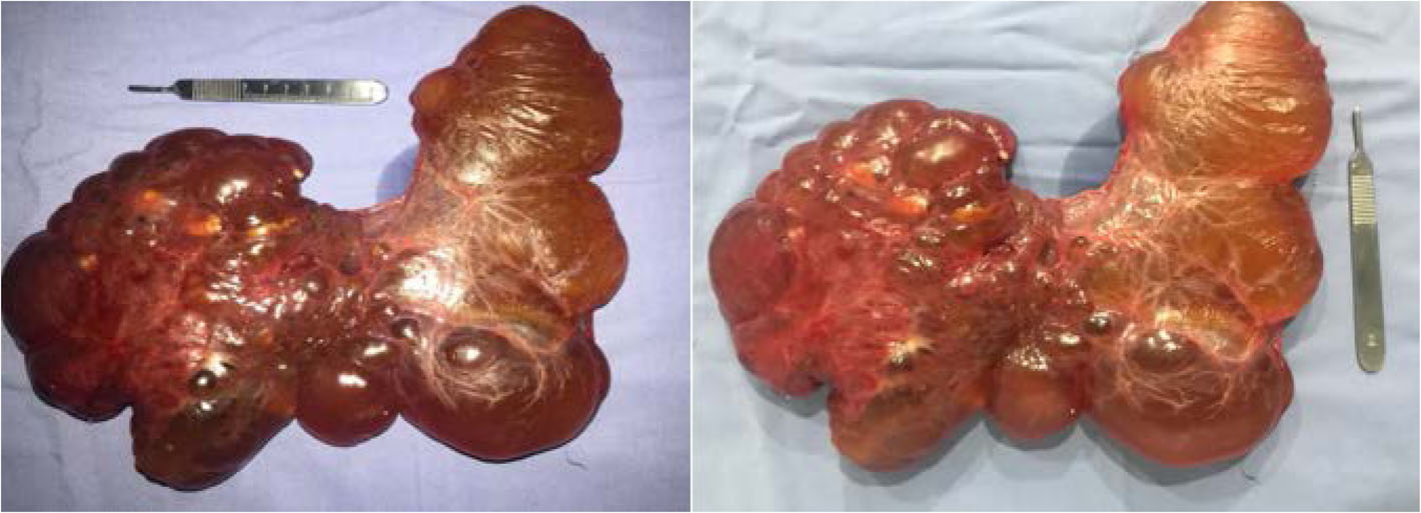
Cystic lesion measuring 30 cm in diameter and weighing approximately 1 kg

Follow-up tests were requested, since the patient presented signs of peritoneal irritation, which found leukocytosis, neutrophilia, and thrombocytosis, PCR (C-reactive protein) indicative of bacterial infection; previously requested blood cultures were negative. An exploratory laparotomy was carried out on suspicion of sepsis of abdominal origin which found a cystic lesion dependent on the greater and lesser omenta, attached to the greater curvature of the stomach and transverse colon, with greenish-yellow content and whitish particles inside, measuring approximately 30 cm in diameter and weighing approximately 1 kg (Fig. 3).

The microscopic description of the pathology showed soft tissues with spaces that appeared to be lymphatic vessels associated with multiple lymphoid aggregates. Septal connective tissue was seen with frequent necrosis. There was no malignancy in this material.

After the postoperative period, the patient adequately tolerated oral administration. The surgical wound was in good condition and progressed adequately, so the patient was discharged with follow-up by pediatric surgery.

## Discussion and conclusions

Mesenteric cysts are benign tumors of congenital origin that predominantly occur during childhood [10]. The most accepted explanation for their etiology is a proliferation of ectopic lymphatic vessels in the mesentery without communication with the lymphatic circulation, which may form anywhere from the mesentery to the retroperitoneum [8, 11].

They can be classified into 4 types according to their morphology: type 1 or pediculate, which is easily resectable; type 2 or sessile (between the 2 layers of the mesentery) requires resection of the affected loops and anastomosis; type 3, which can extend to the retroperitoneum and cannot be completely resected; and type 4, which is multicentric and requires several surgeries and sometimes sclerotherapy [8, 12–15].

These lesions can be asymptomatic and this is the reason for the incidental findings. In the case of our patient, the initial diagnostic approach of the remission site was an acute diarrheal disease, due to the multiple diarrheal episodes presented by the patient, and the associated fever; however, a large abdominal mass was palpated during the physical examination, and diagnostic images were requested.

Ultrasonography of the abdomen is the diagnostic imaging of choice, and it is possible to observe a delimited mass with thin walls and cystic appearance. The findings on an x-ray of the abdomen consist of a homogeneous mass with a density similar to that of the water that displaces the intestinal loops. Magnetic resonance and computed tomography do not provide any information in addition to that which was mentioned for the other diagnostic imaging techniques [8, 11].

In the case of our patient, abdominal radiography and abdominal ultrasound were performed when an abdominal mass was identified upon palpation, which showed displacement of the intestinal loops to the left and opacification of the right hemiabdomen, and a multiseptate image that occupied the right hemiabdomen and displaced the bowel loops, respectively.

These types of masses must be handled surgically and the resectability of the mass depends on its type. Emergency surgical management was considered in our patient’s case, since the patient had abdominal distension associated with symptoms of systemic inflammatory response from the time of admission, and for this reason, sepsis of abdominal origin was considered.

It is important to know these pathologies even though they are infrequent, because although they are benign masses by definition, they can lead to complications such as intestinal torsion, intestinal obstruction, and even peritonitis.
